# Upregulation of CIP2A in estrogen depletion‐resistant breast cancer cells treated with low‐dose everolimus

**DOI:** 10.1002/2211-5463.12956

**Published:** 2020-09-03

**Authors:** Eiji Nishio, Takanori Hayashi, Mao Akaza, Yukiko Hisatomi, Masahiro Hikichi, Takuma Fujii, Toshiaki Utsumi, Nobuhiro Harada, Yohei Shimono

**Affiliations:** ^1^ Department of Obstetrics and Gynecology Fujita Health University School of Medicine Toyoake Japan; ^2^ Department of Biochemistry Fujita Health University School of Medicine Toyoake Japan; ^3^ Department of Breast Surgery Fujita Health University School of Medicine Toyoake Japan

**Keywords:** Akt, breast cancer, CIP2A, estrogen receptor, everolimus

## Abstract

Everolimus (EVE), an inhibitor of mammalian target of rapamycin, is an emerging second‐line therapeutic option for hormone therapy‐resistant breast cancers. However, some patients do not respond to EVE, whereas in others it exacerbates the disease. Cellular inhibitor of protein phosphatase 2A (CIP2A) is a human oncoprotein that can promote cancer cell growth and apoptosis resistance. Although CIP2A is upregulated in hormone‐related cancers, such as breast cancer, little is known about potential anti‐tumor effects of downregulating CIP2A. As a model to study the resistance of breast cancer cells to hormone treatment, we previously established clones of long‐term estrogen depletion‐resistant MCF‐7 (LTED) cells. Here, we selected three clones highly responsive to EVE and three clones poorly responsive to EVE. When cells were treated with EVE, *CIP2A* mRNA expression was decreased in highly responsive EVE clones (DC‐cells) whereas it was increased in poorly responsive EVE clones (IC‐cells). Using Kaplan–Meier survival plots, we report that high expression of CIP2A was associated with significantly reduced overall survival in patients with luminal A breast cancer. In IC‐cells, cell growth was enhanced upon EVE treatment whereas an EVE range of 0.1–100 nm decreased growth in DC‐cells. The mRNA expression of genes involved in epithelial–mesenchymal transition (EMT) such as *CDH1*, *CLDN3*, and *CK19* was significantly decreased in IC‐cells, but remained unchanged in DC‐cells. These findings highlight a relationship between CIP2A and EMT in the intrinsic resistance of hormone therapy‐resistant breast cancers to EVE.

## Graphical abstract

Everolimus (EVE) is a drug that improves resistance to hormone therapy but may have adverse effects on EVE‐resistant patients. We indicated that even low concentrations of EVE could increase CIP2A expression in EVE‐resistant breast cancer cells. High CIP2A expression was associated with reduced luminal A breast cancer patient survival. Moreover, the expression of epithelial–mesenchymal transition‐related genes decreased in EVE‐resistant cells.

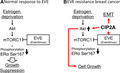

**Abbreviations**
AIs: Aromatase inhibitors
CIP2A: Cellular inhibitor of protein phosphatase 2A
DC‐cells: Highly responsive EVE LTED clones
EMT: Epithelial–mesenchymal transition
EVE: Everolimus
IC‐cells: Poorly responsive EVE LTED clones
LTED: Long‐term estrogen depletion‐resistant MCF‐7

Breast cancer is one of the most common malignancies in women, with an incidence of 2.1 million cases and 0.63 million deaths worldwide in 2018 [1]. It is classified into four types according to the status of expression of hormone receptors. The most common type is the estrogen receptor alpha (ERα)‐positive breast cancer, called the ‘luminal type’. Estrogen plays a crucial role in the development and progression of ERα‐positive breast cancers [2, 3].

Aromatase inhibitors (AIs) which target aromatase, an enzyme involved in the conversion of androgen into estrogen in the body, is a commonly used therapeutic option as hormone treatment (HT) [4, 5, 6, 7, 8] in patients with ERα‐positive breast cancers. AIs generally have fewer side effects and significantly improved efficacy when compared to the currently used breast cancer treatments. Much of the recent research on AIs focuses on determining the mechanisms of resistance to these inhibitors [9, 10, 11, 12].

One of the major reasons why ER‐positive breast cancer develops resistance to AIs is the abnormal activation of ER. The estrogen‐independent aberrant activation of ERα has been shown to occur upon its phosphorylation at serine 167 (S167) through the phosphatidylinositol 3‐kinase‐Akt‐mTOR signaling pathway [13, 14, 15]. Everolimus (EVE), an inhibitor of mammalian target of rapamycin (mTOR), is commonly used to treat HT‐resistant breast cancers [13, 14, 15, 16, 17]. The Breast Cancer Trials of Oral Everolimus‐2 (BOLERO‐2) study showed that the addition of EVE to estrogen depletion therapy significantly improved progression‐free survival, with observed medians of 6.9 and 2.8 months. The results of the BOLERO2 trial demonstrated that EVE improves the treatment of HT‐resistant breast cancer [18].

As a model to study the resistance of breast cancer cells to HT, we previously established 30 clones of long‐term estrogen depletion‐resistant MCF‐7 (LTED) cells. In these cells, we found that the effects of an Akt activator, cellular inhibitor of protein phosphatase 2A (CIP2A), and those of EVE strongly correlated with each other [18, 19]. CIP2A inactivates protein phosphatase 2A (PP2A), which upregulates Akt [20]. It is also a characteristic human oncoprotein that can promote the growth of cancer cells, anchorage‐independent cell growth, and apoptosis resistance [21, 22]. PP2A is frequently inactivated in human cancers, resulting in the induction of epithelial–mesenchymal transition (EMT) and reduction of E‐cadherin and p53 expression [21]. Although the expression of CIP2A is upregulated in hormone‐related cancers, such as breast cancer, little is known about the effects of specific molecular targeted therapy on the expression of CIP2A.

Our previous studies showed that the proliferative capacity of Highly responsive EVE LTED clones (DC‐cells) was suppressed at low concentrations of EVE (IC_50_ < 1.5 nm), whereas that of Poorly responsive EVE LTED clones (IC‐cells) was not affected even at high EVE concentrations (IC_50_ > 200 nm) [19]. In this study, we investigated whether low concentrations EVE (0.01 nm) affect the proliferation of three strains of LTED cells in which CIP2A was increased (increased CIP2A cells: IC‐cells) and three strains in which CIP2A was decreased (decreased CIP2A cells: DC‐cells).

## Materials and methods

### Cell culture

MCF‐7 cells (human ERα‐positive breast cancer cells) were obtained from the American Type Culture Collection (Manassas, VA, USA) and cultured as described previously [19]. To avoid the hormonal effects of phenol red and serum, cells treated with 17 beta‐estradiol (E2) were cultured in phenol red‐free RPMI 1640 medium supplemented with 10% dextran‐coated charcoal‐treated FBS (Nichirei Biosciences, Inc., Tokyo, Japan). LTED cells, which model estrogen depletion‐resistant cells, were established as described previously [23, 24, 25].

### Cell proliferation assay

The viability of cultured cells was determined using a CCK‐8 kit (Dojindo Molecular Technologies, Kumamoto, Japan) to evaluate the effect of EVE (0.01, 0.1, 1, 10, and 100 nm, Wako Pure Chemical Industries, Ltd., Osaka, Japan) on LTED cells as described previously [19]. Briefly, cells (1000 cells/well) were seeded in 96‐well plates and cultured in medium supplemented with one or more drugs at 37 °C in a 5% CO_2_ incubator for 96 h.

### Western blotting

Whole‐cell lysates were prepared using lysis buffer (62.5 mm Tris/HCl, pH 6.8, 5% 2‐mercaptoethanol, 2% sodium dodecyl sulfate, 5% sucrose, and 0.01% bromophenol blue; Wako Pure Chemical Industries, Ltd.), and the concentration of proteins in the lysates was measured using a DCTM Protein Assay kit (Bio‐Rad Laboratories, Inc., Hercules, CA, USA) as described previously [19]. The samples (5 μg protein/lane) were separated by electrophoresis on 10% SDS/PAGE and transferred to polyvinylidene difluoride (PVDF) membranes (GE Healthcare, Piscataway, NJ, USA). The PVDF membranes were processed as described previously [19]. The antibodies used were rabbit polyclonal and monoclonal antibodies against Akt (1 : 2000 dilution), phosphorylated Akt Ser473 (1 : 1000 dilution), Rb (1 : 1000 dilution), phosphorylated Rb (1 : 1000 dilution), and CIP2A (1 : 1000 dilution) (all obtained from Cell Signaling Technology, Inc., Danvers, MA, USA), and horseradish peroxidase‐labeled secondary anti‐rabbit (1 : 10 000 dilution; Bio‐Rad Laboratories) or anti‐mouse (1 : 5000 dilution; MBL, Nagoya, Japan) antibodies. The intensity of the chemiluminescence of specific bands was quantified using cool saver software (ATTO, Tokyo, Japan). Rabbit polyclonal antibodies against GAPDH (1 : 2000 dilution) were purchased from Santa Cruz Biotechnology (Santa Cruz Biotechnology, Dallas, TX, USA). All antibodies were diluted in Can Get Signal® Immunoreaction Enhancer Solution (Toyobo, Inc., Osaka, Japan).

### Immunofluorescence analysis

Long‐term estrogen depletion‐resistant MCF‐7 cells were seeded on cover slips and allowed to adhere overnight. After incubation with DMSO or EVE (100 nm) for 72 h, the cells were fixed with 4% paraformaldehyde in PBS, permeabilized in 0.1% Tween 20/PBS, and blocked with 1% BSA. The cells were washed and incubated with anti‐human E‐cadherin (1 : 100 dilution), cytokeratin 19 (CK19, 1 : 100 dilution), and ZEB1 (1 : 50 dilution) antibodies (all obtained from Cell Signaling Technology, Inc.) for 2 h at room temperature. The cells were subsequently washed and incubated with Alexa488‐labeled anti‐rat secondary antibody (Invitrogen, Carlsbad, CA, USA) for 2 h. After staining the nuclei with DAPI, the cells were visualized using a fluorescence microscope, BZ‐9000 (Keyence Corporation, Osaka, Japan).

### RNA extraction and quantitative PCR

Total RNA was extracted from treated cells using TRIzol reagent (Qiagen, Hilden, Germany) and reverse‐transcribed using the PrimeScript RT reagent kit (Takara Bio, Inc., Shiga, Japan). Quantitative PCR was performed in triplicate using the ABI Perkin‐Elmer Prism 7300HT Sequence detection system (Applied Biosystems, Foster City, CA, USA). TaqMan gene expression assays (Applied Biosystems) were used to detect the expression of *CIP2A* (TaqMan Accession ID Hs00405413_m1), *CDH1* (Hs999999905_m1), *CLDN3* (Hs999999905_m1), *CK19* (Hs999999905_m1), and *ZEB1* (Hs999999905_m1); *GAPDH* (Hs999999905_m1) was used as an internal control gene. Relative expression levels were determined using the ΔΔC*_t_* method, according to the manufacturer's instructions.

### Statistical analysis

Results were analyzed for statistical significance using one‐way analysis of variance followed by Fisher's protected least significant difference test when the data were derived from more than two groups. For significance tests based on three groups or more, subsequent analysis with a *post hoc* test with the Bonferroni correction was performed. The paired Student's *t*‐test was performed where indicated, and *P*‐values of < 0.05 were considered statistically significant.

## Results

### CIP2A is a predictor of poor prognosis in patients with ER‐positive, HER2‐negative breast cancer

**Fig. 1:**
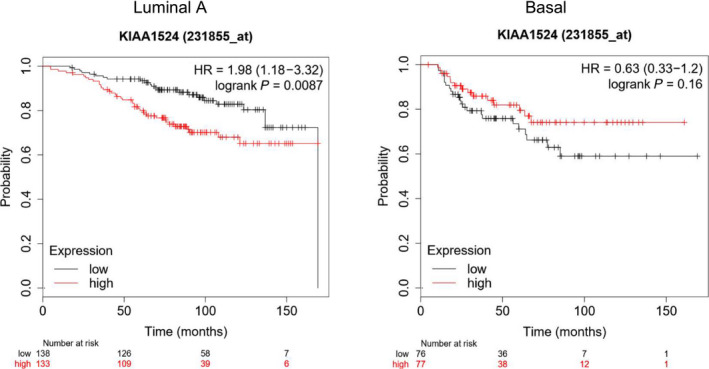
Effects of *KIAA1524* (CIP2A) on survival rates of breast cancer patients. A Kaplan–Meier plotter database was used to analyze the prognostic significance of *KIAA1524* (CIP2A) in luminal A (*n* = 271) and basal (*n* = 153) breast cancer. The red lines signify individuals with high expression of *KIAA1524* (CIP2A), and black lines indicate those with low expression.

To analyze the effects of CIP2A expression in patients with ER‐positive breast cancer, we used the Kaplan–Meier plotter database (http://kmplot.com/), which is commonly used in cancer research [26, 27], to predict prognostic value. *CIP2A* is referred to as *KIAA1524* in the Kaplan–Meier plotter (Fig. 1). The overall survival (OS) of 153 patients with basal‐like (ER‐/HER2−) and 271 patients with luminal A (ER+/HER2−) breast cancer was analyzed using the Kaplan–Meier survival plot. High expression of *CIP2A* was associated with significantly reduced OS in patients with luminal A breast cancer [hazard ratio = 1.98 (1.18–3.32), *P* < 0.01] but not in patients with basal‐like breast cancer [hazard ratio = 0.63 (0.33–1.20), *P* = 0.156]. These results suggest that the relationship between *CIP2A* expression and therapeutic effect might be specific for ERα‐positive breast cancer.

### Low doses of EVE increase the expression of CIP2A in EVE‐resistant breast cancer cells

We previously established 30 clones of LTED cells and performed drug sensitivity assays to determine the half‐maximal inhibitory concentration (IC_50_) for EVE in each clone [18, 19]. To further evaluate the effect of EVE on CIP2A mRNA expression, we selected three LTED clones (DC‐cells: clone number L3, L5, and L22) highly responsive to EVE and three clones (IC‐cells: clone number L4, L7, and L29) poorly responsive to EVE.

**Fig. 2:**
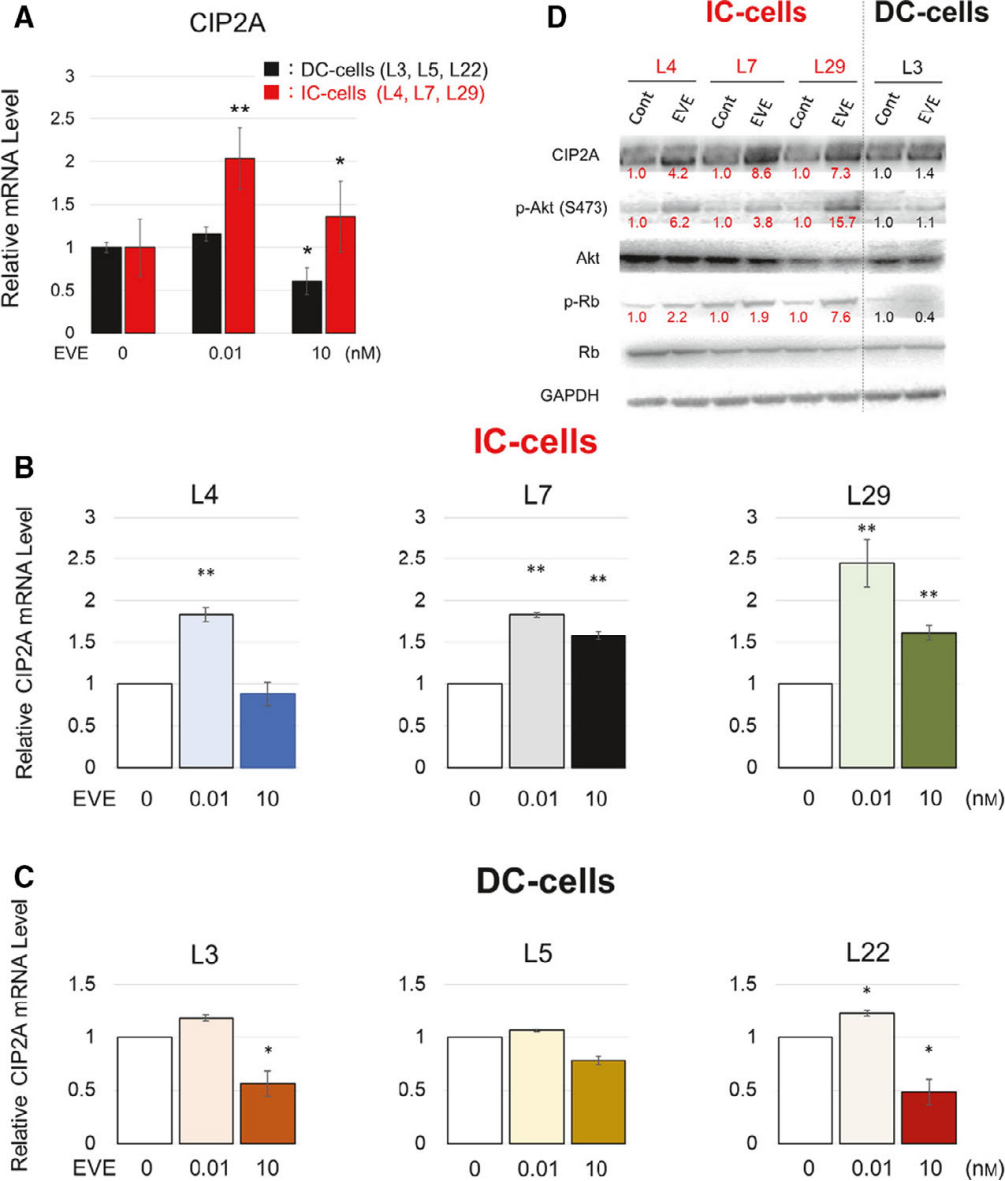
Effect of EVE on the expression of CIP2A. DC‐cells (L3, L5, and 22, black bars) and IC‐cells (4, 7, and 29, red bars) were incubated with 0.01 and 10 nm EVE. The medium was changed every 2 days. The number of the cells was measured with a CCK‐8 kit. The relative mRNA levels of CIP2A were evaluated in DC‐ and IC‐cells treated with EVE at the concentrations indicated. GAPDH was used for normalization. (A) Average of DC‐ and IC‐cells (*n* = 3 per treatment group). (B) L3, L5, and L22 (*n* = 3 per treatment group). (C) L4, L7, and L29 (*n* = 3 per treatment group). (D) Western blot analysis of the expression of CIP2A, pAkt (S473), Akt, Rb, and phosphorylated Rb. The image represents cropped areas of the polyvinylidene fluoride membrane, with each area indicating the reactivity of the indicated antibody. The relative value to the band intensity of the control is shown in the figure. GAPDH was used as a loading control (*n* = 3 per treatment group). Data represent means ± SD of three independent experiments (Student's *t*‐test; *P < 0.05; **P < 0.01).

These cells were incubated with or without EVE (0.01 or 10 nm) for 72 h. The mRNA expression levels of *CIP2A* were significantly increased in cells that were poorly responsive to EVE and treated with 0.01 nm EVE (Fig. 2A). The relative expression levels of CIP2A in each LTED cell line exposed to EVE are shown in Fig. 2B,C. CIP2A levels in three LTED clones that were highly responsive to EVE were reduced by treatment with 10 nm EVE. This result is consistent with the findings of our previous study [13]. However, this experiment revealed that low concentrations of EVE (0.01 nm EVE) increased the expression of *CIP2A* in poorly responsive LTED cells. Therefore, the three clones in which *CIP2A* expression was increased by EVE were referred to as IC‐cells, whereas the three clones in which CIP2A expression was increased by EVE were indicated as DC‐cells. Next, we examined whether cells with increased CIP2A expression activated the Akt signaling and consequently affected the phosphorylation of Rb. The level of CIP2A in IC‐cells was increased by 0.01 nm EVE. In these IC‐cells, the levels of both phosphorylated Akt and phosphorylated Rb were increased (Fig. 2D).

### Low concentrations of EVE increase the proliferation of IC‐cells

**Fig. 3:**
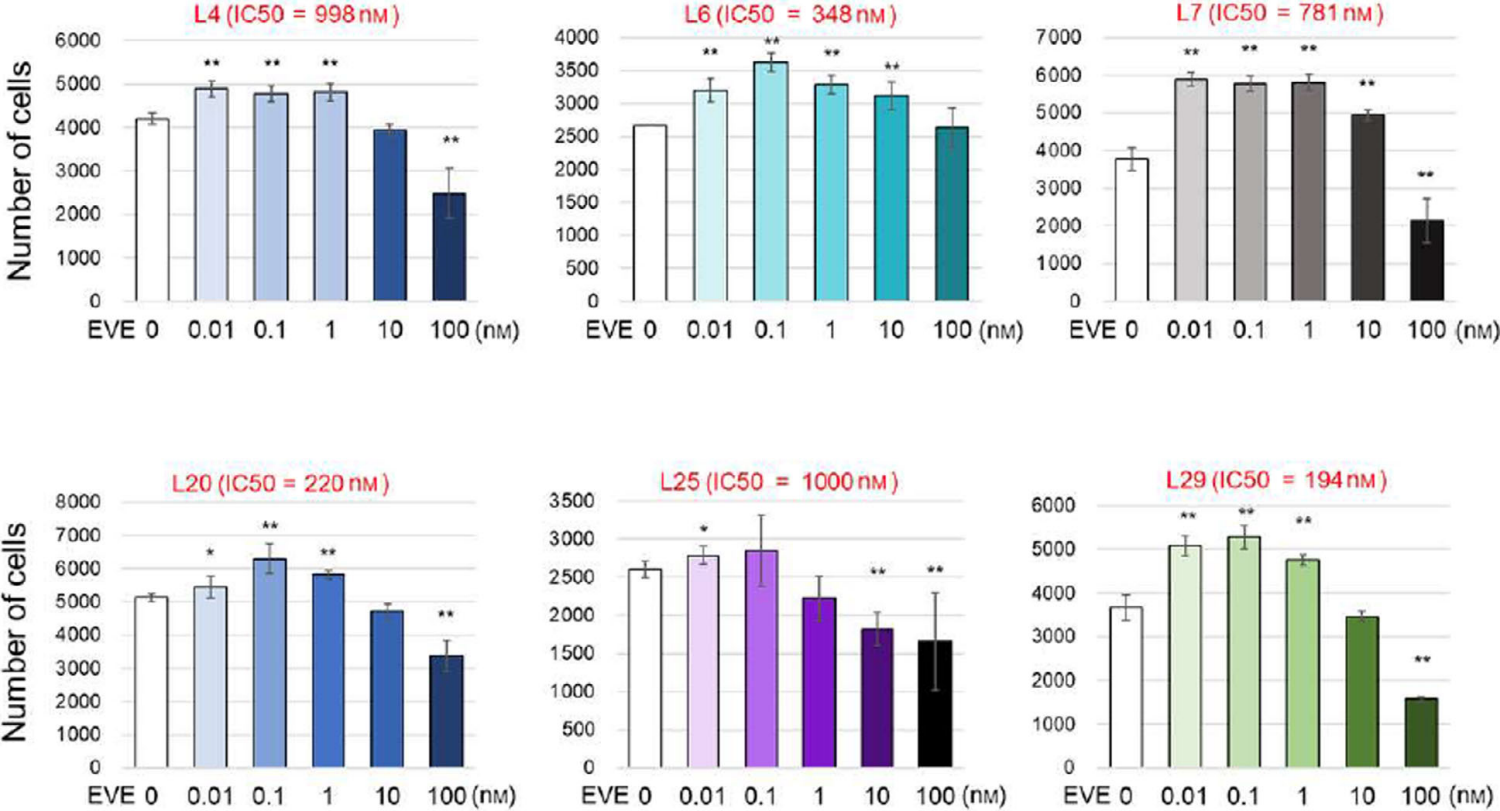
Effect of low concentrations of EVE on the proliferation of IC‐cells. The viability of cells with poor response to EVE when exposed to EVE was assessed; L4, L6, L7, L20, L25, and L29 are LTED clones with an IC_50_ higher than 100 nm. These clones were incubated with or without EVE (0.01, 0.1, 1, and 100 nm). The medium was changed every 2 days. The number of cells was measured with a CCK‐8 kit (*n* = 3 per treatment group). Data represent means ± SD of three independent experiments (Student's *t*‐test; *P < 0.05; **P < 0.01).

**Fig. 4:**
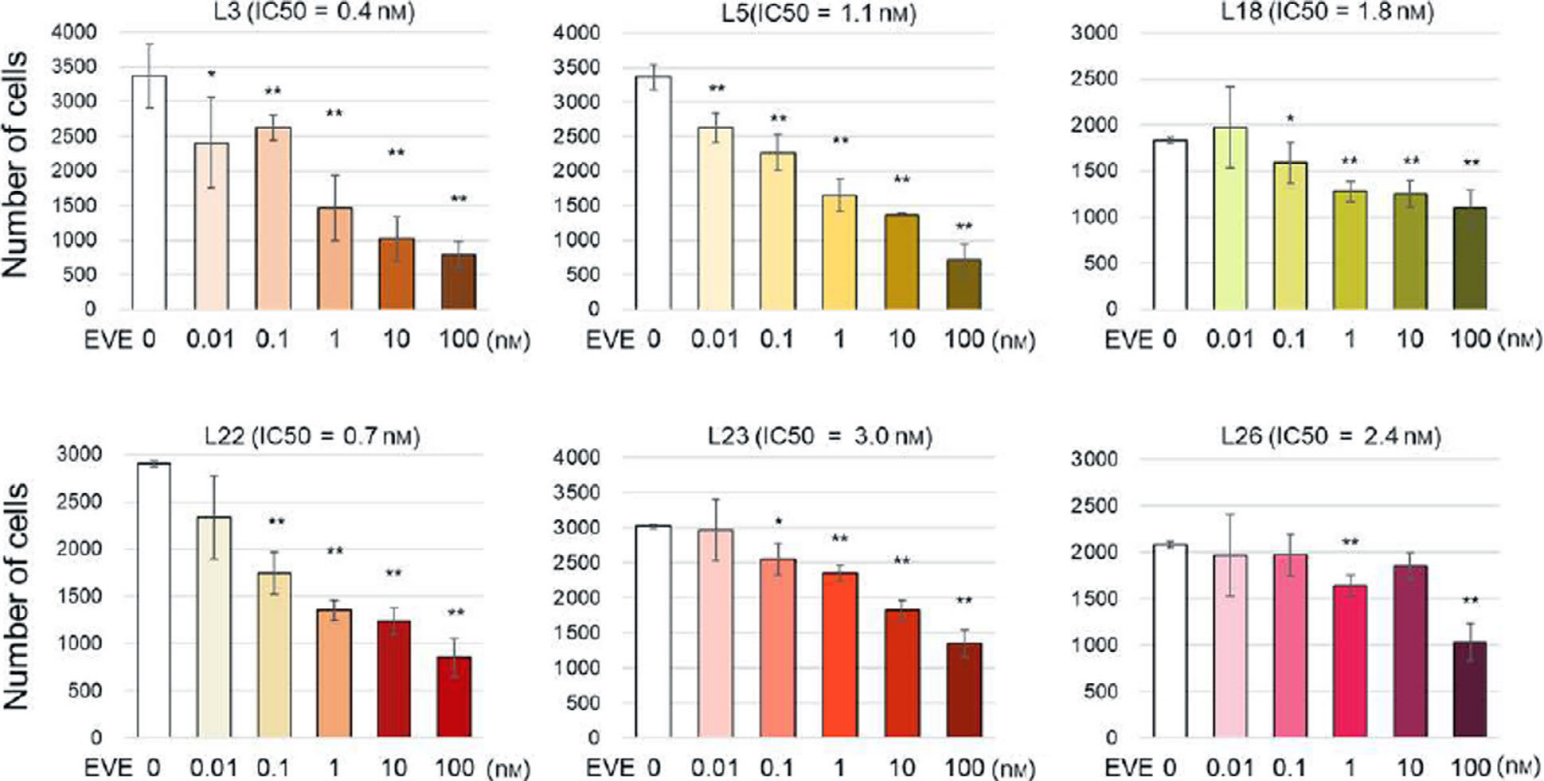
Effect of low concentrations of EVE on the proliferation of DC‐cells. The viability of cells highly responsive to EVE was assessed when exposed to EVE; L3, L5, L18, L22, L23, and L26 are LTED clones, with an IC_50_ lower than 5 nm. These clones were incubated with or without EVE (0.01, 0.1, 1, and 100 nm). The medium was changed every 2 days. The number of cells was measured with a CCK‐8 kit (*n* = 3 per treatment group). Data represent means ± SD of three independent experiments (Student's *t*‐test; *P < 0.05; **P < 0.01).

Because higher expression levels of *CIP2A* were associated with poorer prognosis and low concentrations of EVE‐activated Akt signaling in IC‐cells (Figs 1 and 2), we next analyzed whether EVE increases the proliferation of IC‐cell clones [19]. Both IC‐ and DC‐cells were incubated with or without EVE (0.01, 0.1, 1, and 100 nm) for 4 days. The number of cells increased significantly when the cells were exposed to 0.01, 0.1, and 1 nm EVE (Fig. 3). In contrast, in the clones that were highly responsive to EVE, the number did not increase when the cells were exposed to 0.01 nm EVE and it rather decreased when 0.1–100 nm EVE was added (Fig. 4).

### Low concentrations of EVE enhance EMT in IC‐cells

**Fig. 5:**
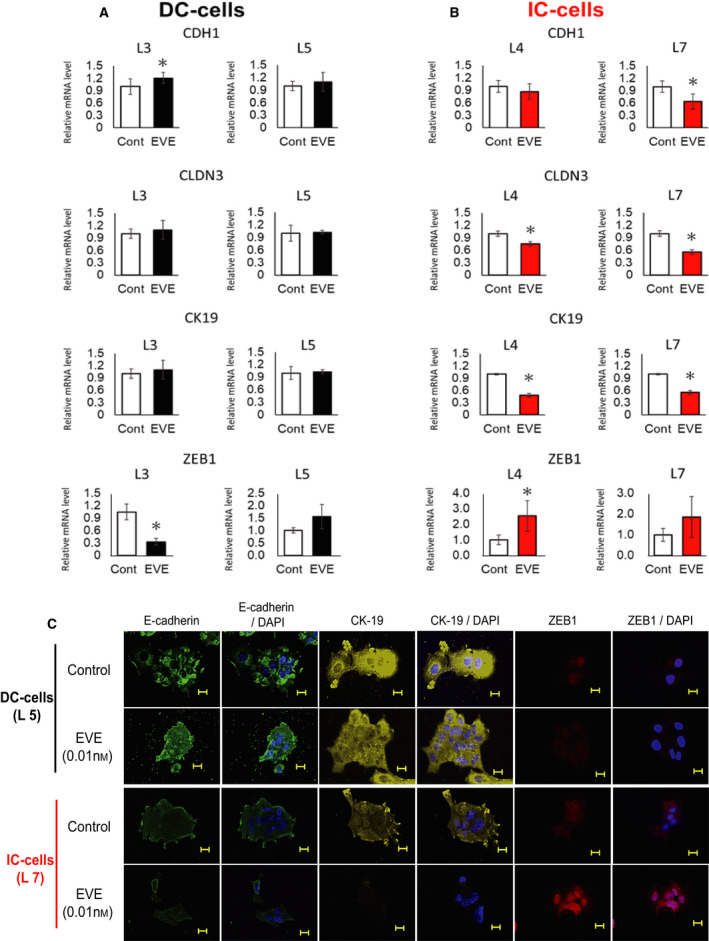
Effect of low concentrations of EVE on EMT in DC‐ and IC‐cells. Clone numbers L3 and L5 (indicated as DC‐cells) and L7 and L4 (indicated as IC‐cells) were assessed. DC‐ and IC‐cells were incubated with or without EVE (0.01 nm) for 48 h. The relative mRNA levels of *CDH1*, *CLDN3*, and *CK19* were evaluated in DC‐ and IC‐cells treated with EVE at the concentrations indicated. *GAPDH* was used for normalization. (A) mRNA expression levels of *CDH1*, *CLDN3*, and *CK19* in L3 and L5 cells (*n* = 3 per treatment group). Data represent means ± SD of three independent experiments (Student's *t*‐test; *P < 0.05). (B) mRNA expression levels of *CDH1*, *CLDN3*, and *CK19* in L4 and L7 cells (*n* = 3 per treatment group). Data represent means ± SD of three independent experiments (Student's *t*‐test; * P < 0.05). (C) Expression levels of E‐cadherin, CK19, and ZEB1 in DC‐cells (L5) and IC‐cells (L7). Representative pictures of tumor cells and EVE are shown (*n* = 1). Scale bar: 20 μm.

CIP2A is known to promote EMT by reducing the number of epithelial cancer cells [20, 21]. We selected two clones that were highly responsive to EVE, DC‐cells (clone numbers L3 and L5), and two clones that were poorly responsive to EVE. IC‐cells (clone numbers L4 and L7). The cells were incubated with or without EVE (0.01 nm) for 48 h. There was no significant change in the mRNA expression levels of *CDH1*, *CLDN3*, or *CK19* in DC‐cells (Fig. 5A). In contrast, the mRNA expression levels of *CDH1*, *CLDN3*, and *CK19* were significantly decreased in all IC‐cells (Fig. 5B).

To further confirm the effect of EVE (0.01 nm) on EMT, the expression of E‐cadherin, CK19, and ZEB1 in DC‐cells (L5) and IC‐cells (L7) was verified using immunofluorescent staining (Fig. 5C). ZEB1 is expressed in most human cancers in epithelial tissues. EVE treatment decreased the expression of E‐cadherin and CK19 and increased the expression of ZEB1 in IC‐cells (L7). In contrast, E‐cadherin in DC‐cells (L5) was slightly increased by EVE treatment.

## Discussion

**Fig. 6:**
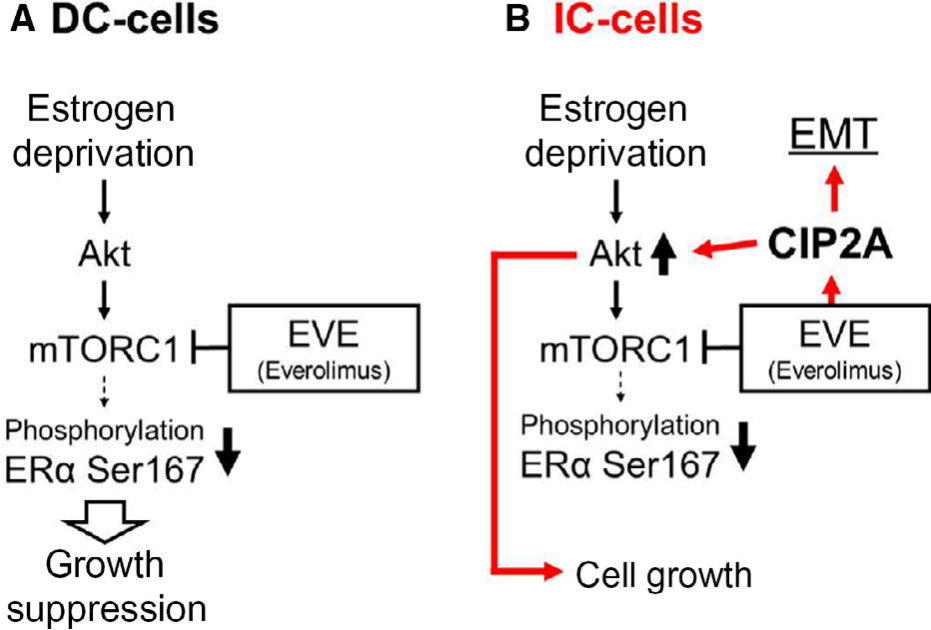
Effects of low‐dose EVE on the signaling pathways assessed in the present study. (A) Schematic diagram of the activated Akt pathway in estrogen deprivation‐resistant breast cancer. One of the mechanisms underlying estrogen deprivation resistance is the aberrant activation of ERα, dependent on its phosphorylation at S167, through the Akt–mTOR signaling pathway, which regulates several cellular functions including cell growth and survival. Treatment with the mTOR inhibitor, EVE, eliminates estrogen deprivation therapy‐resistant breast cancer cell growth. (B) In all EVE‐resistant LTED clones (IC‐cells) investigated, low‐dose EVE increased the expression of CIP2A, activated the Akt signaling and enhanced the cell growth. Although EMT was enhanced, the molecular mechanism remains unclear. EVE‐activated pathways revealed in this study are indicated using red lines.

Everolimus is a molecular targeting drug developed to overcome HT resistance. However, 15% of cancers are EVE‐resistant at the beginning of treatment [28]. Previous studies using multiple LTEDs showed that EVE increases the expression levels of CIP2A and that CIP2A‐mediated activation of Akt signaling counteracts the effect of EVE [19, 20, 21, 22]. EVE is inhibited *in vitro* by pS6 kinase in a concentration‐dependent manner (IC_50_: 0.1–100 nm) [28]. Therefore, it is orally administered at a maximum dose of 10 mg (0.1 mmol) per day. It was also determined that this drug acts at a concentration of 0.3 nm or less in the patient’s body. In this study, we found that in EVE‐resistant clones (IC‐cells), even very low concentrations (0.01–1 nm) of EVE increased the expression of CIP2A, which in turn induced the phosphorylation of Rb. Using six LTED clones, we observed that minimal concentrations (0.01–100 nm) of EVE promoted cell growth (Fig. 3). Moreover, our data suggest that EVE induces EMT and proliferation in EVE‐resistant breast cancer. This study was conducted based on the concentration of EVE used by patients. Therefore, the enhanced EMT observed in IC‐cells may occur in patients with EVE resistance (Fig. 6).

CIP2A is a novel oncoprotein driving the malignant phenotype through and has been reported to be overexpressed in 39% of breast cancer cases [29]. It has also emerged as a neoplastic protein that is generally overexpressed in many tumors and correlates with higher tumor grade and resistance to treatment. CIP2A also induces EMT and is associated with drug resistance [21]. Moreover, the expression of CIP2A may be useful as a novel prognostic and predictive indicator of tamoxifen resistance and recurrence in ER‐positive breast cancer [30]. Our findings indicate that EVE can cause disease progression via CIP2A, suggesting that CIP2A can be used as a potential biomarker to optimize treatment of HT‐resistant breast cancers.

## Conflict of interest

The authors declare no conflict of interest.

## Author contributions

Most of the experiments were performed by TH. EN, MA, YH, and MH conducted the experiments and performed the data analysis. YS, NH, TU, and TF participated in the designing of the experiments and supervised the study. All authors read and approved the final manuscript.

## Data Availability

The datasets used and/or analyzed during the current study are available from the corresponding author upon reasonable request.
